# A computational pipeline to discover potential cross-reactive antibodies: a case study on coronavirus

**DOI:** 10.3389/fcimb.2026.1692727

**Published:** 2026-02-09

**Authors:** Li Ye, Mengdie Hu, Yuan Wang, Zihao Li, Yewei Cun, Kailin Tang, Zhiwei Cao

**Affiliations:** 1 School of Life Sciences and Technology, Tongji University, Shanghai, China; 2 School of Life Sciences, Fudan University, Shanghai, China

**Keywords:** conserved surface site, coronavirus, cross-reactive antibody, epitope prediction, SARS-CoV-2, virtual screening

## Abstract

**Introduction:**

Cross-reactive antibody (crAb), which recognizes conserved viral epitopes across diverse strains, has emerged as powerful tools for diagnostics, therapeutic targeting, and pandemic preparedness. Yet conventional crAb discovery primarily relies on hybridoma technology and phage display which are time-consuming and labor-intensive.

**Methods:**

Here, we present an integrative computational pipeline that leverages conformational epitope prediction and epitope immunogenic similarity analysis to compute cross-reactive epitopes, so as to prioritize potential crAbs undisclosed before.

**Results:**

Taking coronavirus as an example, we demonstrate that this pipeline successfully predicted candidate crAbs for five coronavirus antigens, including SARS-CoV-2 variants (WT, Beta, Omicron B.1, XBB.1.5) and one SARS-CoV variant. Out of Top20 predicted candidates, 45% were validated in CoV-AbDab as cross-reactive across coronavirus variants, including experimentally confirmed crAbs such as P17 (IC_50_ = 0.165 ng/mL against SARS-CoV-2 WT), BG7-15 (IC_50_ = 16 ng/mL against SARS-CoV-2 WT), and Beta-54 (IC_50_ = 1 ng/mL against Gamma variant).

**Discussion:**

Though in pilot-study, this pipeline might serve as a scalable and efficient strategy for rapidly prioritizing potential crAbs in research of infectious disease.

## 1 Introduction

Cross-reactive antibodies (crAbs), defined by their ability to recognize conserved epitopes shared across antigenically distinct variants, represent a critical component of immune defense against rapidly evolving viral pathogens (Reche, 2020). Unlike strain-specific antibodies, crAbs provide intrinsic robustness against viral mutation and antigenic drift, making them highly valuable for diagnostics, immune surveillance, and pandemic preparedness. Among which, broadly neutralizing antibodies (bnAbs) constitute a functionally elite subset that exhibits neutralizing activity against multiple strains, typically through high-affinity binding to evolutionarily constrained sites (Martinez et al., 2021; Chen et al., 2023). In addition to serving as prospecting pools for bnAb discovery, crAbs can also exert protective effects through synergistic mechanisms such as Fc-mediated effector functions (Bournazos et al., 2020). This endows crAbs with strong potential for proactive defense against zoonotic spillover and viral escape, highlighting their indispensable role in next-generation antiviral therapeutics.

Conventional approaches to crAb discovery, such as hybridoma technology and phage display, typically rely on infected or immunized hosts for the screening of immune B cells or library construction. These methods have successfully yielded crAbs against coronaviruses, such as the cross-neutralizing antibodies 7D6 and 6D6 derived from mice sequentially immunized with SARS-CoV-2, SARS-CoV, and MERS-CoV spike proteins (Li et al., 2021). Broadly reactive monoclonal antibodies binding have been reported with shared motifs across SARS-CoV-2, hCoV-OC43, and hCoV-HKU1. Moreover, several animal coronaviruses have also been isolated through phage display (Klompus et al., 2021). In recent years, a comprehensive database CoV-AbDab (Raybould et al., 2021) has been systematically curated collecting many experimentally validated antibodies, which integrates sequence, structural, and functional annotations with cross-reactive properties for coronavirus. While these experimental efforts provide reliable crAbs, computational methods are highly desirable to predict potential crAbs from a pool of binder candidates to save the time and cost.

So far, literature review showed that only sparse computational methods have been developed to directly compute and prioritize crAbs (Lou et al., 2023; Williams et al., 2025), although a number of methods can predict conserved epitope regions (Shehata et al., 2021; An et al., 2022; Kaushik et al., 2022; Afshari et al., 2023). A pioneering effort in this direction is XBCR-net (Lou et al., 2023), a deep learning framework designed to predict broadly reactive antibodies directly from B-cell receptor sequences. XBCR-net employs an atrous convolutional neural network to extract sequence features from antibody variable regions and antigen sequences, followed by a multilayer prediction module to estimate antibody binding probabilities across multiple viral variants. This sequence-based approach demonstrated impressive predictive performance in identifying antibodies with experimentally validated cross-reactivity against SARS-CoV-2 variants, highlighting the feasibility of antibody-centric crAb prediction.

As is well recognized, antibodies predominantly recognize surface-exposed epitopes on antigens, and over 80% of antibody epitopes, including those targeted by crAbs, are conformational or structural in nature rather than linear (Kumar et al., 2023). These considerations motivate us to explore whether incorporating conserved conformational epitopes and structure interfacial information can improve the accuracy and interpretability of crAb prediction, particularly across antigen variants with substantial sequence divergence.

Here, we present an integrative, structure-based computational pipeline for systematic discovery of cross-reactive antibodies. Our framework is specifically designed to rank existing antibodies according to their potential to recognize structurally conserved, cross-reactive epitopes (CREs) across multiple antigen variants. By integrating conformational epitope prediction, conserved surface site analysis, and epitope immunogenic similarity comparison, the pipeline enables virtual screening of large antibody libraries for crAb candidates while simultaneously suggesting the structural determinants of cross-reactivity.

Taking coronaviruses as a case study, we applied this pipeline to screen the antibody library for a panel of spike protein antigens from SARS-CoV and multiple SARS-CoV-2 variants (WT, Beta, Omicron B.1, and XBB.1.5). Coronaviruses represent an ideal testbed for crAb study due to their rapid antigenic evolution in recent years coupled with the presence of structurally constrained regions on the spike glycoprotein, which is the primary target of neutralizing and cross-reactive antibodies (Lauring and Hodcroft, 2021; Sharma et al., 2022). This combination of sequence divergence and structural conservation provides a stringent and biologically relevant setting to evaluate structure-guided crAb screening strategies.

## 2 Methods

### 2.1 Data preparation

Antibody sequences used for screening crAbs were obtained from the CoV-AbDab database (12,916 antibodies) (Raybould et al., 2021). We retained only entries with paired heavy and light chains and performed sequence de-duplication based on 100% identity across the full-length V(D)J regions. This produced a non-redundant dataset of 10,231 paired antibody sequences for downstream analyses.

To obtain a sequence-diverse subset suitable for large-scale virtual screening, we further clustered the non-redundant antibody set using CD-HIT (Li et al., 2001). The sequence identity threshold and the alignment coverage threshold for longer and shorter sequences are all set to 80% for CD-HIT. One representative antibody was selected from each cluster, resulting in 1,273 antibodies for structural modeling and epitope-based screening.

### 2.2 Antibody structure modeling

Structures for the 1,273 representative antibodies were modeled using AbodyBuilder2 (Dunbar et al., 2016) with default parameters, focusing on the Fv region. All sequences were numbered using the IMGT scheme, ensuring consistent definition of framework and CDR regions across antibodies and enabling standardized downstream mapping of predicted epitope–paratope interfaces.

### 2.3 Antigen structure preparation

Spike protein structures for five coronavirus variants were collected from the Protein Data Bank (PDB) (Berman et al., 2000): SARS-CoV (PDB: 5X5B), SARS-CoV-2 WT (7Z3Z), Beta (7WEV), Omicron B.1 (7QO7), and XBB.1.5 (8V0T).

To ensure comparability of epitope accessibility across variants, we standardized all antigens to an RBD-up spike conformation. Specifically, when multiple PDB entries were available, we selected spike trimers in which the receptor-binding domain (RBD) adopts an “up” state, as this conformation represents the receptor-accessible prefusion state and exposes RBD surface patches that are frequently targeted by cross-reactive antibodies (Gui et al., 2017; Yang and Rao, 2021).

### 2.4 Epitope area prediction

For each antibody candidate, we predicted its potential binding epitope residues on each spike antigen using the SEPPA-mAb platform (Qiu et al., 2023) (online access: http://www.badd-cao.net/seppa-mab/). SEPPA-mAb is an antibody-specific conformational epitope prediction method that evaluates the shape complementarity, chemical properties, and other local features of antigen surface regions to estimate their likelihood of being recognized by a given antibody.

For each antigen, SEPPA-mAb represents antigen surface patches and the corresponding antibody CDRs as structural and physicochemical fingerprints. These fingerprints are used to compute a binding score between each surface patch and CDR, reflecting the predicted interaction propensity. To determine whether one residue is an epitope residue or not, the residue score for any residue r can be calculated and adjusted by the following equations:


$$raw\_residue\_score_{r}=\;\frac{\sum \frac{1}{1+d}\;*PC\_score_{i}}{M}$$

$$adjust\_residue\_score_{r}=\;\frac{\sum raw\_residue\_score_{j}}{N}$$

where $PC\_score_{i}$ represents the predicted PC score of surface patch i which contains residue r, d is the distance of residue r to the center of patch i, M is the total number of patches, which contains residue r, and $\sum raw\_residue\_score_{j}$ represents the sum of the raw residue score of all neighboring surface residues within 5 Å atom distance of target residue r, whereas N means the total number of the above residues.

Finally, residues with SEPPA-mAb binding probability ≥0.392 were defined as predicted epitope residues. Residues surpassing this threshold for each variant were integrated to form the potential binding epitope for downstream conservation and cross-reactivity analyses.

### 2.5 Conserved surface site identification

To identify conserved surface sites (CSS), we combined sequence conservation from multiple sequence alignment (MSA) with structure-based solvent accessibility. We first aligned the spike protein sequences from the five coronavirus variants (SARS-CoV, SARS-CoV-2 WT, Beta, Omicron B.1, and XBB.1.5) using Clustal Omega (Sievers et al., 2011) and quantitatively assessed site-wise conservation from the alignment output. Residues identical in 100% of the aligned sequences were defined as conserved sites; this criterion can be relaxed depending on the number of antigens considered and their phylogenetic distances.

We then identified surface-exposed residues by calculating solvent accessible surface area (SASA) for each antigen structure using Biopython’s Shrake–Rupley implementation (Ribeiro et al., 2019), with a standard water probe radius of 1.4 Å. Residues with SASA > 1 Å² were considered surface-exposed and retained. Integrating the MSA-derived conserved residues with the SASA-derived surface residues yielded the CSS set for each variant, and CSSs shared across all five variants were defined as common CSSs for downstream epitope conservation analysis.

### 2.6 Conserved surface site percentage

To quantify the sequence conservation of predicted conformational epitopes across variants, we introduced the metric common CSS percentage of epitope. For a specified epitope i from one pair of variant and antibody, the common CSS percentage of epitope can be calculated by the following equation:


$$common\_CSS\_percentage\_of\_epitope_{i}=\;\frac{\sum CSS_{j}}{N_{i}}$$

where $\sum CSS_{j}$ represents the sum of all CSS within the epitope i, whereas $N_{i}$ represents the total number of epitope residues.

This metric provided a structural basis for assessing the degree of reactivity and conservation within epitope regions across variants. We focused on top-ranking antigen–antibody combinations exhibiting high common CSS percentage of epitope, which are more likely to retain structural compatibility across variants.

### 2.7 Cross-reactive epitope evaluation and crAbs prioritization

We then calculated potential cross-reactive epitopes by assessing immunogenicity similarity across variants using CE-BLAST (Qiu et al., 2018), which integrates physicochemical and structural features to compute similarity scores between conformational epitopes. For each retained antibody, we calculated pairwise CE-BLAST scores among epitopes predicted on different variant antigens. Epitope pairs with CE-BLAST scores exceeding empirical cutoffs (0.8 within SARS-CoV-2 variants; 0.4 across SARS-CoV and SARS-CoV-2) were defined as candidate CREs, and antibodies targeting these CREs were designated as crAbs.

Finally, we split epitope evaluation into two groups, within-virus and across-virus, filtered candidates using group-specific support thresholds (≥0.35 within-virus; ≥0.15 across-virus), and then ranked antibodies separately within each group by the maximum CE-BLAST score among the corresponding variant pairs.

## 3 Results

### 3.1 Pipeline construction

The computational pipeline to discover potential crAbs encompasses three steps: (i) predicting conformational epitopes for each antibody candidate and retaining only those with a converged epitope area across different antigen variants. In other words, find those antibodies with a relatively converged binding area on different antigen structures; (ii) identifying common conserved surface sites (CSS) within each predicted epitope area across antigen variants and retaining only those antibodies with epitope area containing high percentage CSS; (iii) calculating immunogenicity similarity among conformational epitopes across variants and ranking the antibody by these similarity scores. In general, the higher the similarity score among those conformational epitopes, the higher the possibility that the antibody will be cross-reactive across variants.

**Figure 1:**
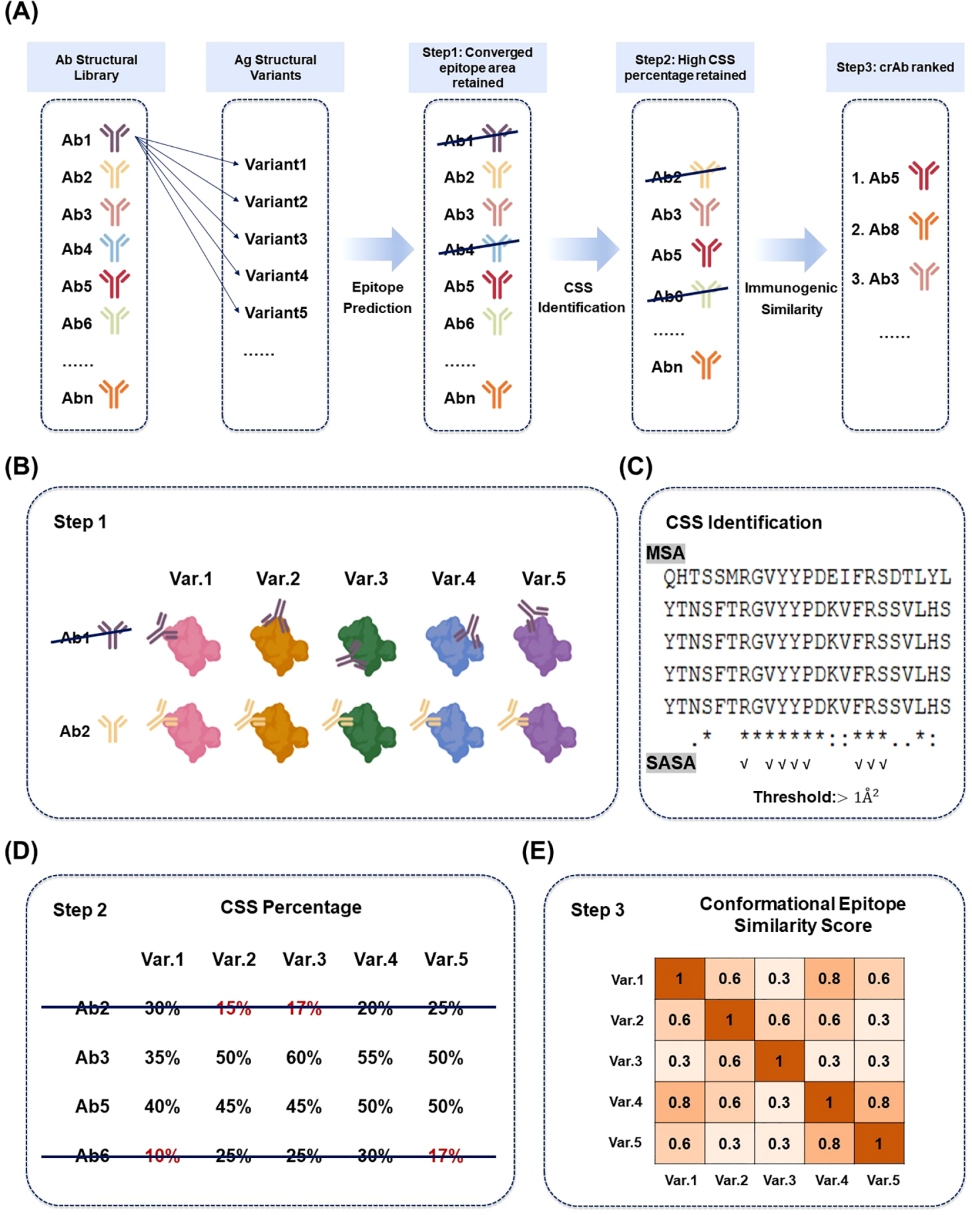
**(A)** Pipeline to discover potential crAbs. **(B)** In step 1, SEPPA-mAb was adopted to predict conformational epitopes for a given antibody structure, and we retained those Abs with converged epitope area. **(C)** Conserved surface site (CSS) identification was conducted by multiple sequence alignment (MSA) and solvent-accessible surface area (SASA) calculation. “*” indicates that the amino acids at this position are identical across all sequences (“:” indicates strong similarity, “.” indicates weak similarity). **(D)** In step 2, the Abs with high CSS percentages within the predicted epitopes were remained. **(E)** In step 3, CE-BLAST was used to calculate the epitope similarity between different variants and obtain the ranking list of potential crAbs according to CE-BLAST scores.

The workflow is illustrated in **Figure 1**. In the first step, we excluded antibodies predicted to bind to markedly different surface regions on different antigen variants. As the variants usually belong to one antigen family and have highly similar structures, crAbs are expected to bind to the same surface area across different variant structures. The freely available tool SEPPA-mAb was firstly utilized to predict epitope area for each antibody (Qiu et al., 2023). SEPPA-mAb was purposely designed to predict conformational epitope for antibody binders. Those antibodies will be excluded if their epitope areas are found to be inconsistent across variants.

In the second step, we further filtered out antibodies whose predicted epitopes showed substantial differences in residue composition across variants. Because epitopes enriched in conserved amino acids are more likely to elicit crAbs, antibodies targeting unconserved epitopes at the sequence level were removed to reduce computational cost. We performed multiple sequence alignment (MSA) across variants to identify conserved sites and defined conserved surface sites (CSS) as conserved residues that are solvent-accessible based on solvent-accessible surface area (SASA) analysis. Only antibodies whose predicted epitopes contained a high proportion of CSS were retained for downstream analysis.

Based on step 2, we conducted further conservation analysis by immunogenicity similarity at the conformational level for high-CSS epitopes. Even in the same surface area and the same epitope positions, the detailed residues may vary to different extents across individual variants due to rapid mutation and evolution. Therefore, sequence conservational analysis may not be sensitive enough to detect epitopes with similar immunogenicity bound by the same antibody. CE-BLAST (conformational Epitope -Basic Local Alignment) was here employed for this purpose, which has been previously used to suggest the potential cross-reactive conformational epitopes between dengue virus and zika virus, as well as between SARS-CoV and SARS-CoV-2 (Qiu et al., 2020; Zhao et al., 2024). The ranking list of potential crAbs can be obtained according to their similarity scores of conformational epitopes among the variants.

### 3.2 Identification of cross-binding epitopes and crAbs for coronavirus

Taking coronavirus as an example, we applied the above pipeline to screen a library of 1,273 representative antibody sequences derived from CoV-AbDab, against five spike antigen variants, namely, SARS-CoV (PDB ID: 5X5B) SARS-CoV-2 wild type (PDB ID: 7Z3Z) and SARS-CoV-2 variants of Beta (PDB ID: 7WEV), Omicron B.1 (PDB ID: 7QO7), and XBB.1.5 (PDB ID: 8V0T).

**Figure 2:**
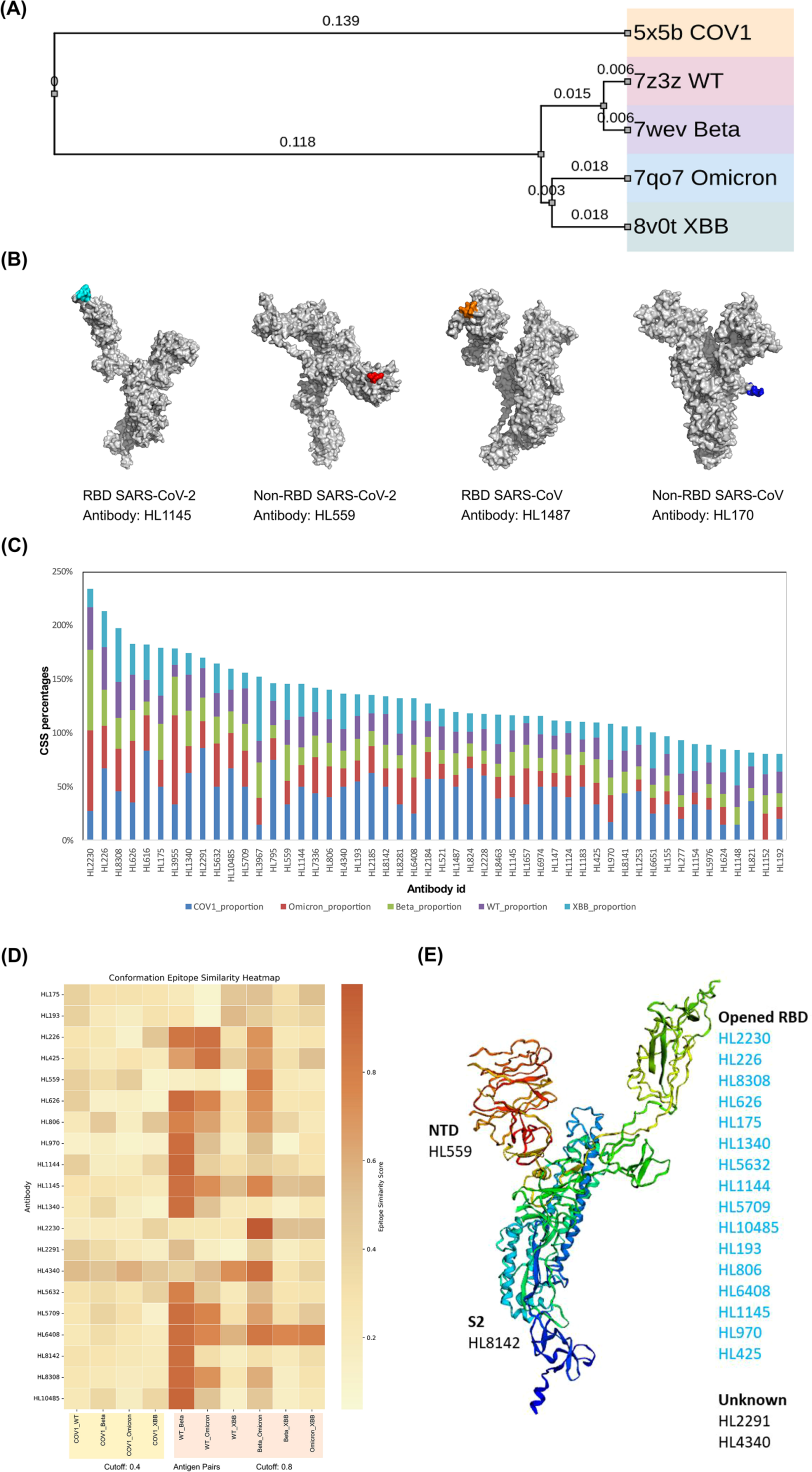
**(A)** Phylogenetic tree illustrating the phylogenetic distance between the five virus strains, plotted by iTOL v6 (Letunic and Bork, 2024). Branch lengths correspond to amino acid substitutions per site, with numerical values indicating evolutionary distances. **(B)** Representative 3D visualizations of predicted epitope residues (highlighted regions) in the RBD and non-RBD regions of the spike protein for SARS-CoV-2 and SARS-CoV. **(C)** Common CSS percentage of epitope among the five virus variants for the 50 candidate antibodies. The stacked bar chart illustrates the proportion of the CSS percentage contributed by each of the five variants—COV1 (SARS-CoV, light blue), Omicron (red), Beta (green), WT (purple), and XBB (teal)—for the 50 antibody candidates. **(D)** Heatmap showing the conformation epitope similarity of different antibodies obtained using CE-BLAST. The similarity cutoff is 0.8 within SARS-CoV-2 virus (light coral region) and 0.4 across viruses (light orange region). **(E)** The binding regions of the top 20 predicted antibodies (validated by the CoV-AbDab database). The antibodies whose actual binding regions aligned with predicted regions are displayed in cyan-highlighted text.

As shown in **Figure 2A**, different SARS-CoV-2 variants exhibited close phylogenetic distances and they clustered far from SARS-CoV. **Figure 2B** illustrates examples of epitope prediction results of step 1 from SEPPA-mAb for individual variants. Each converged epitope area was derived for remaining antibodies across variants after step 1.

At step 2, 668 common conserved surface sites (CSSs) were initially obtained across all five antigens. The common CSS percentage of epitope was calculated for each individual variant, and 50 antibodies were remained with CSS percentage over 20% in each epitope area of all variants (**Supplementary Table S1**). The overall CSS-percentage distribution across different variants is displayed in **Figure 2C** for all the 50 antibodies.

At step 3, pair-wised CE-BLAST scores were calculated among the five variants for each of the 50 antibodies. According to the empirical cutoff (a maximum score above 0.8 within a virus or 0.4 across viruses), a total of 20 potential crAbs were remained as the most promising ones (total unique crAbs from **Tables 1**, **2**), with CE-BLAST scores being displayed in a heat map in **Figure 2D**. Research data of these 20 antibodies showed that their epitope areas are located within the N-terminal domain (NTD), the receptor-binding domain (RBD), and the S2 subunit (Du et al., 2009; Sinha et al., 2023), with a predominant enrichment observed in the RBD region (**Figure 2E**). Following filtering, final ranking was performed separately for the within-virus and across-virus groups which were divided by the group-specific minimum support thresholds (≥0.35 within-virus; ≥0.15 across-virus) and we ranked candidates by the maximum CE-BLAST similarity among the corresponding variant pairs. This dual-control strategy ensures both the breadth of the reaction spectrum and the potential for cross-reactive binding.

**Table 1:** Ranking list of potential candidate antibodies within SARS-CoV2.

Rank	Ab ID	Max score	Ab name	Known binding profiles
1	HL2230	0.99	BD-821	SARS-CoV2_WT
**2**	**HL10485**	**0.93**	**N12-9**	**SARS-CoV2_WT; SARS-CoV2_Omicron**
**3**	**HL1144**	**0.92**	**Beta-54**	**SARS-CoV2_WT; SARS-CoV2_Beta;** **SARS-CoV2_Omicron; SARS-CoV2_Omicron-XBB**
**4**	**HL626**	**0.9**	**BG7-15**	**SARS-CoV; SARS-CoV2_WT; SARS-CoV2_Omicron**
5	HL970	0.9	C2456	SARS-CoV2_WT
**6**	**HL8308**	**0.9**	**S5D2**	**SARS-CoV2_WT; SARS-CoV2_Beta**
7	HL1340	0.89	WRAIR-2151	SARS-CoV2_WT
8	HL5709	0.89	BD-821	SARS-CoV2_WT
9	HL8142	0.89	PGT-128	SARS-CoV2_WT
**10**	**HL1145**	**0.88**	**Beta-44**	**SARS-CoV2_WT; SARS-CoV2_Beta**
11	HL4340	0.88	368.02a.C.0248	SARS-CoV2_WT
12	HL6408	0.88	C3085	SARS-CoV2_WT
**13**	**HL226**	**0.87**	**P17**	**SARS-CoV; SARS-CoV2_WT; SARS-CoV2_Omicron**
**14**	**HL425**	**0.86**	**XG005**	**SARS-CoV2_WT; SARS-CoV2_Beta**
15	HL559	0.81	S2-L28	SARS-CoV2_WT

Bold values highlight antibodies proved to be capable of cross-reacting with our target antigens.

**Table 2:** Ranking list of potential candidate antibodies across SARS-CoV2 and SARS-CoV.

Rank	Ab ID	Max score	Ab name	Known binding profiles
1	HL4340	0.59	368.02a.C.0248	SARS-CoV2_WT
**2**	**HL226**	**0.48**	**P17**	**SARS-CoV; SARS-CoV2_WT; SARS-CoV2_Omicron**
3	HL806	0.48	7B8	SARS-CoV2_WT
**4**	**HL2291**	**0.44**	**CC84.1**	**SARS-CoV; SARS-CoV2_WT**
5	HL559	0.43	S2-L28	SARS-CoV2_WT
**6**	**HL626**	**0.43**	**BG7-15**	**SARS-CoV; SARS-CoV2_WT; SARS-CoV2_Omicron**
**7**	**HL1144**	**0.43**	**Beta-54**	**SARS-CoV2_WT; SARS-CoV2_Beta;** **SARS-CoV2_Omicron; SARS-CoV2_Omicron-XBB**
8	HL5632	0.42	AB-2125	SARS-CoV2_WT
**9**	**HL193**	**0.41**	**C032**	**SARS-CoV; SARS-CoV2_WT**
10	HL175	0.41	Fab2-4	SARS-CoV2_WT

Bold values highlight antibodies proved to be capable of cross-reacting with our target antigens.

### 3.3 Pipeline assessment and peer comparison

To assess the predictive accuracy of our computational pipeline, we validated the 20 antibody candidates against experimental binding data from the CoV-AbDab database (Raybould et al., 2021). CoV-AbDab contains relevant metadata including evidence of cross-reactivity, epitope region (specific to functional region), and sourcing literatures.

Firstly, by comparing the predicted epitope area with the experimentally confirmed epitope information from the CoV-AbDab database, we found that 16 antibodies were confirmed to target the same domain of RBD we predicted as marked in cyan color (**Figure 2E**), indicating the accuracy of the epitope prediction. Among the 20 antibodies, 9 were experimentally confirmed as crAbs according to CoV-AbDab information. This overall 45% success rate underscores the predictive power of our pipeline. Then, two ranking lists were made according to CE-BLAST scores. One for within-virus of SARS-CoV-2 (**Table 1**), and another for cross-virus of SARS-CoV-2 and SARS-CoV (**Table 2**).

Secondly, among the 20 antibodies, those with the maximum epitope similarity within a virus greater than 0.8 can be grouped into a list containing 15 candidate antibodies (**Table 1**). Examining antibodies of within-virus ranking list showed a positive correlation between high similarity and broader cross-reactive breadth within SARS-CoV-2 (**Table 1**). For example, HL1144 (ranked 3) was found to bind nearly all known SARS-CoV-2 variants tested, including recent Omicron subvariants such as XBB.1.5 and EG.5. HL8308 (ranked 6) was proved to bind with WT, Alpha, Beta, and Delta. HL1145 (ranked 10) demonstrated confirmed binding to a wide range of SARS-CoV-2 variants, including WT, Alpha, Beta, and Gamma. HL425 (ranked 14) was also proved to bind with WT, Alpha, Beta, Gamma, and Delta. In total, the majority of antibodies with CE-BLAST scores above 0.9 had multiple supporting entries in CoV-AbDab, validating the pipeline in predicting variant-level cross-reactivity. These results indicate that antibodies with high epitope similarity are more likely to remain effective across virus mutants.

Using a criterion of greater than 0.4 for the maximum epitope similarity across viruses, we obtained a cross-virus ranking list containing 10 antibodies (**Table 2**). Examining the cross-virus ranking list showed a similar conclusion that can further validate the predictive value of our method. Among the top ranking, HL226 (ranked 2) and HL193 (ranked 9) demonstrated cross-binding to SARS-CoV, supporting their potential as pan-sarbecovirus antibodies. HL2291 (ranked 4) showed binding to SARS-CoV-2 and SARS-CoV. HL626 (ranked 6) was also proved to bind both SARS-CoV-2 variants and SARS-CoV, as well as other coronaviruses like Pangolin-GD. The confirmed epitope domains for 80% of predicted antibodies, coupled with experimental cross-reactivity for 45%, especially those with broad within-virus and cross-virus activity, highlight the utility of this pipeline for guiding the discovery of next-generation crAbs across evolving coronavirus variants.

Comparing the performance of our pipeline with peers, we applied XBCR-net (Lou et al., 2023) to the same dataset of 1,273 representative antibodies against the five Spike antigens. The top 20 antibodies predicted by XBCR-net are summarized in **Table 3**. Among the 20, XBCR-net successfully suggested five crAbs with a positive rate of 25%. In contrast, our pipeline yielded nine crAbs out of 20, with a positive rate of 45%. Compared with XBCR-net, the enrichment ability got enhanced by 80% in our pipeline. Although XBCR-net is faster, our pipeline gives more validated positives and additionally provides informative epitope positions for each predicted crAb. Interestingly, there was no overlap between the two sets of top 20 candidates, suggesting that the two approaches might be complementary to some extent.

**Table 3:** Ranking list of potential crAbs predicted by XBCR-net.

Rank	Ab ID	Average score across five antigens	Ab name	Known binding profiles
1	HL1520	0.9995	GAR16	SARS-CoV2_WT
2	HL683	0.9992	C868	SARS-CoV2_WT
3	HL347	0.9982	C530	SARS-CoV2_WT
4	HL7076	0.9981	CQTS163	SARS-CoV2_WT
**5**	**HL1966**	**0.9980**	**X01**	**SARS-CoV; SARS-CoV2_WT; SARS-CoV2_Beta**
6	HL6566	0.9978	C999	SARS-CoV2_WT
7	HL744	0.9978	C954	SARS-CoV2_WT
8	HL7917	0.9978	MERS-14	MERS-CoV
9	HL9432	0.9977	BD56-1682	SARS-CoV2_Omicron
10	HL325	0.9977	C067	SARS-CoV2_WT
11	HL369	0.9974	C096	SARS-CoV2_WT
**12**	**HL7248**	**0.9973**	**DH1101**	**SARS-CoV; SARS-CoV2_WT**
13	HL3971	0.9966	23E	SARS-CoV
14	HL7925	0.9965	MERS-7	MERS-CoV
15	HL7547	0.9957	H6	SARS-CoV2_WT
16	HL7929	0.9943	MERS-93	MERS-CoV
17	HL10209	0.9940	BD55-6469	SARS-CoV2_WT
**18**	**HL580**	**0.9928**	**P008_076**	**SARS-CoV; SARS-CoV2_WT**
**19**	**HL3493**	**0.9917**	**BD56-208**	**SARS-CoV; SARS-CoV2_WT; SARS-CoV2_Omicron; SARS-CoV2_Omicron-XBB**
**20**	**HL2300**	**0.9882**	**CC84.10**	**SARS-CoV; SARS-CoV2_WT; SARS-CoV2_Beta; SARS-CoV2_Omicron**

Bold values highlight antibodies proved to be capable of cross-reacting with our target antigens.

Collectively, these validation and comparison results from the public domain underscore the effectiveness and advantages of our pipeline in prioritizing potential crAbs.

### 3.4 Case studies of top candidate antibodies

To further evaluate the accuracy of our prediction pipeline, we examined three top-ranked antibody candidates, P17, BG7-15, and Beta-54. These antibodies have previously been characterized for their cross-reactivity profiles against multiple SARS-CoV-2 variants and SARS-CoV, enabling a comparison between experimentally validated binding predictions and our computational predictions.

**Figure 3:**
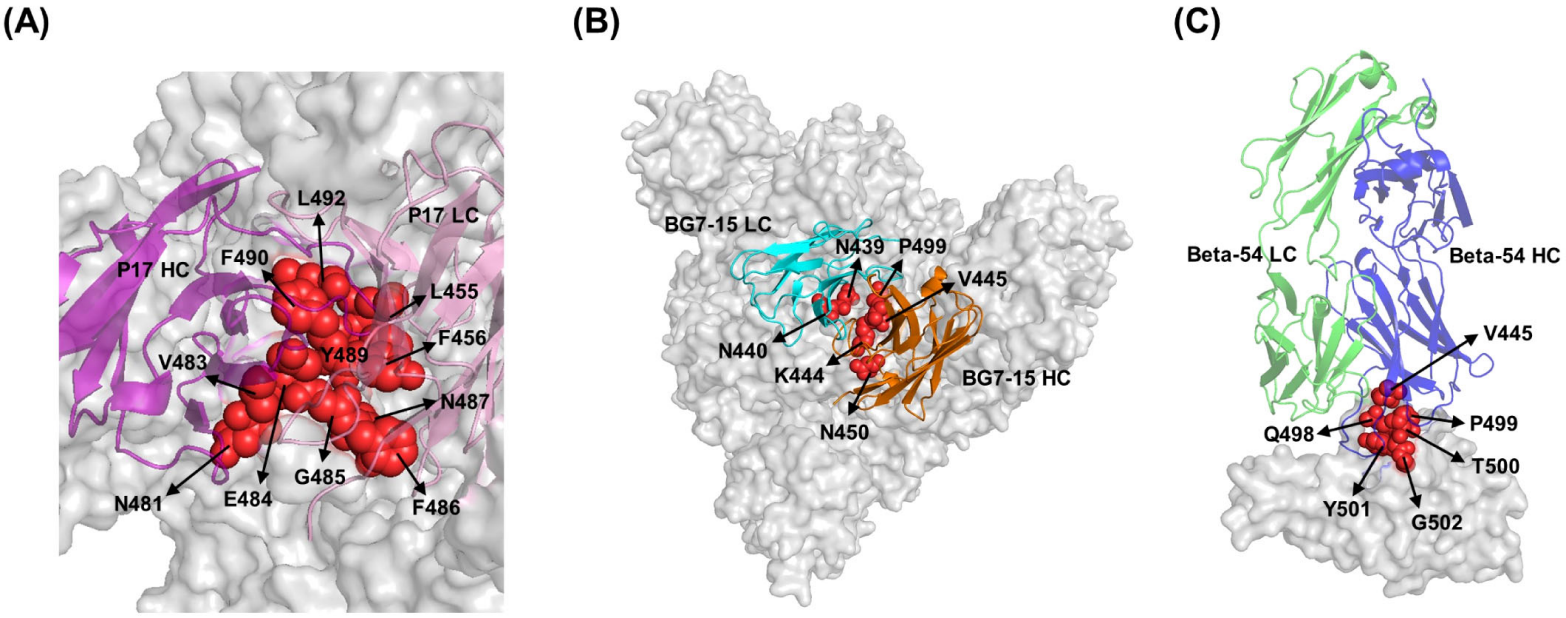
Structural illustration of antibody binding to the spike protein. Residues comprising the epitopes are shown as spheres and colored in red. **(A)** Binding interface representations of P17 and SARS-CoV-2. The heavy and light chains are shown as cartoon and colored in purple and pink, respectively. **(B)** Binding interface representations of the BG7–15 and SARS-CoV-2. The heavy and light chains are shown as cartoon and colored in orange and cyan, respectively. **(C)** Binding interface representations of Beta-54 and SARS-CoV-2 S1. The heavy and light chains are shown as cartoon and colored in blue and green, respectively.

P17 (HL226, maximum CE-BLAST score across viruses: 0.48) exhibited a broad binding ability across the SARS-CoV-2 variant panel and SARS-CoV in our computational screen. Previous research has shown that P17 achieves broad SARS-CoV cross-reactivity by targeting a structurally conserved and sterically accessible RBD epitope, leveraging a dense network of hydrophobic and hydrophilic interactions that block ACE2 engagement, and synergizing with companion antibodies to lock the spike protein and prevent viral entry (Yao et al., 2021). In more detail, P17 targets an epitope on the RBD that remains exposed regardless of whether the RBD is in the “closed” or “open” state, allowing it to consistently engage with different structural forms of the spike protein. For the SARS-CoV-2 RBD, SEPPA-mAb prediction localized the P17 binding epitope to residues 484-489. This shows a significant overlap with the literature-verified epitope (residues 455-456, 481, 483-487, 489, 490, and 492) in the core epitope area, demonstrating the accuracy of our pipeline. High-resolution cryo-EM also revealed that P17 binds a conserved patch on the RBM by forming a dense network of hydrophobic (e.g., F486 and Y489) and polar contacts—anchoring the antibody to a site critical for viral entry and conserved across SARS-CoV and SARS-CoV-2 (**Figure 3A**).

The BG7-15 (HL626, maximum CE-BLAST score across viruses: 0.43) antibody, also ranked among the top antibodies in our computational pipeline, exhibits broad reactive activity against multiple SARS-CoV-2 variants and SARS-CoV. BG7–15 is a human monoclonal antibody identified in a study focused on cross-neutralizing responses to both SARS-CoV-2 and SARS-CoV (Scheid et al., 2021). It was originally isolated from a COVID-19 convalescent patient and shown to derive from the IGHV1–18 gene segment, known to contribute to cross-reactive antibody lineages. BG7–15 targets a conserved epitope on the RBD, but importantly, outside the receptor-binding motif (RBM)—the portion directly engaging ACE2. BG7–15 is capable of binding to the “down” RBD conformation (**Figure 3B**) and prevents the RBD from transitioning into the “up” conformation required for receptor binding. The residues contacted by BG7–15 are highly conserved across multiple sarbecoviruses, explaining its broad reactivity against both SARS-CoV-2 variants and SARS-CoV. Structural studies confirmed that mutations present in most variants of concern do not significantly impact its binding.

Moreover, the monoclonal antibody Beta-54 (HL1144, maximum CE-BLAST score within virus: 0.92) demonstrates cross-reactivity within multiple SARS-CoV-2 variants—including the WT, Beta, Omicron, and XBB—in our computational workflow. Beta-54 was originally identified from memory B cells of a COVID-19 convalescent patient who had been infected with the Beta variant of SARS-CoV-2 (Liu et al., 2022). It effectively neutralizes the Omicron variant *in vitro* (Liu et al., 2022) and also retains activity against the Alpha and Gamma variants, although its neutralization potency is reduced against the Victoria and Delta strains (Liu et al., 2022). Structurally, Beta-54 targets a conserved ridge region on the RBD that lies outside the highly variable receptor-binding motif (RBM), thereby avoiding major mutational hotspots such as K417, E484, and N501 (**Figure 3C**). Its binding is facilitated by recognition of conserved structural elements, whereas flexibility in the paratope allows it to accommodate peripheral mutations, including Omicron’s G446S substitution. These features underline its broad neutralization profile across diverse variants.

## 4 Discussion

In this study, we developed and validated a computational pipeline to identify cross-reactive monoclonal antibodies targeting similar epitopes across multiple antigen variants. By integrating conformational epitope prediction and epitope comparison, our pipeline enabled prioritization of crAb candidates with broad binding potential. The advantage lies in its pre-screening of large antibody sequence libraries without prior information of their binding to candidate antigen variants.

Our pipeline covers three steps of filtering including epitope prediction, CSS calculation and immunogenicity comparison. Technically, the conformational epitope could be realized by other tools such as the well-known DiscoTope (Høie et al., 2024), SEMA-3d (Shashkova et al., 2022), and epitope3D (Da Silva et al., 2022). The reason we chose SEPPA-mAb here is that SEPPA-mAb was one of the few freely available tools to predict antibody-specific epitopes. The epitope prediction is enabled by evaluating the physio-chemical complementarity between surface patches of antigen and CDR of antibody. The accuracy achieved 0.873 on 193 independent testing Ab-Ag complexes at the single-residue level, and a false positive rate (FPR) of 0.097 for epitope residue classification, demonstrating significant advantages over docking strategy and AlphaFold (Abramson et al., 2024), particularly on theoretically modeled antibody structures (Qiu et al., 2023).

The purpose of CSS calculation for each epitope area is to pre-select those structurally accessible and sequence conserved surface regions across antigen variants for immunogenic comparison with save computational time, as calculating antigenic similarity for conformational epitopes is computationally costing. Here, we chose the CE-BLAST (Qiu et al., 2018) developed by our group. The CE-BLAST algorithm was purposely designed to compare the antigenic similarity/distance between conformational epitope pairs based on local structure and physicochemical properties via “spin-image” and “shell structure” strategies. Prediction cross-reactivity for 3,867 HA pairs showed an overall AUC of 0.917 on historical experimental data of hemagglutination inhibition assays for influenza A/H3N2 (Qiu et al., 2018). SEPPA-mAb and CE-BLAST scores form an orthogonal assessment to evaluate cross-reactive probability. Users can rank the final list according to different needs of CE-BLAST scores, where we only consider the breadth profile.

From conformational epitope prediction to immunogenicity-similarity calculation, our pipeline effectively boiled down an antibody library over 1,200 to 20 potential candidates. Out of the Top 20 high-confidence candidates, nine were confirmed in CoV-AbDab as cross-reactive across SARS-CoV-2 variants or between SARS-CoV and SARS-CoV-2. The high performance of our pipeline may benefit from integrating enough structural epitope information for synergistic judgment of epitope positions and cross-reactivity, whereas XBCR-net focused on predicting binding probabilities and does not explicitly pinpoint epitope sites that may drive cross-reactivity. Detailed case studies of top candidates such as P17, BG7-15, and Beta-54 provide further confidence in our strategy. In our pipeline, SEPPA-mAb precisely predicted the specific binding positions for P17. For other antibodies in Top ranking, such as C3085(HL6408), 368.02a.C.0248(HL4340), and 7B8(HL806), it is highly worth testing their cross-reactivity systematically though without previous experimental support.

Despite the promising performance of our pipeline, several limitations remain. The accuracy of predictions depends heavily on the quality of structural inputs and the performance of epitope prediction tools, which may not fully account for conformational flexibility, glycosylation, or other post-translational modifications (Raybould et al., 2019). Moreover, a key distinction must be made between cross-reactivity and cross-neutralization—while our approach identifies antibodies likely to bind conserved regions, binding alone does not guarantee protective or inhibitory activity. Future improvements could include glycosylation-aware modeling, the use of deep learning frameworks for affinity and neutralization prediction (Jumper et al., 2021), and the integration of patient-derived antibody repertoires to enhance biological relevance. Ultimately, all *in silico* predictions must be complemented by experimental validation to confirm functional efficacy.

Overall, this study presents a training-free computational pipeline that can be used to prescreen the antibody library for desirable crAbs. Notably, the framework is not restricted to viral targets and can be extended to other antigen families, such as bacteria and other infectious pathogens. In addition to a library of antibodies, it is also applicable to search against a library of well-known antigens with hundreds of strains with increasing input of computational power. In a landscape where rapid response to emerging pathogens is critical, such workflows may contribute to pandemic preparedness by informing the development of broadly neutralizing antibody and broadly protective vaccines.

## 5 Conclusion

This study presents a computational pipeline to facilitate the rapid discovery of crAbs and demonstrates an example on coronavirus spike antigens. By combining structural conservation, binding potential, and conformational epitope similarity scoring, the workflow enables the systematic identification of antibodies capable of recognizing conserved regions across viral variants. The successful identification of candidate antibodies against multiple coronavirus strains—including SARS-CoV-2 and SARS-CoV—highlights the workflow’s potential in guiding broad-spectrum therapeutic development. While experimental validation remains essential, this approach offers a powerful, scalable tool for accelerating antibody discovery and enhancing preparedness for future viral outbreaks.

## Data Availability

The original contributions presented in the study are included in the article/**Supplementary Material**. Further inquiries can be directed to the corresponding authors.
